# How host ecology influences the parasite communities of three Australian flathead fishes, *Platycephalus* spp. (Scorpaeniformes: Platycephalidae)

**DOI:** 10.1007/s00436-024-08359-y

**Published:** 2024-10-03

**Authors:** Owen Bellingham, Tommy L. F. Leung

**Affiliations:** Zoology, School of Environmental and Rural Science, University of New England, Armidale, NSW Australia

**Keywords:** *Platycephalus*, Fishes, Helminths, *Corynosoma*, Parasite community, Host ecology

## Abstract

This study investigated the role of host traits, habitat, and sampling season on the prevalence and intensity of parasites in three species of platycephalid fishes. Three host species sampled were dusky flathead (*Platycephalus fuscus*, *n* = 3), blue-spotted flathead (*Platycephalus caeruleopunctatus*, *n* = 38), and tiger flathead (*Platycephalus richardsoni*, *n* = 59). A total of 14 metazoan parasite species were collected over 15 months, between July 2020 and September 2021. The parasites found included a chondracanthid copepod, adult hemiuroidean trematodes, cestode plerocercoids, larval and adult acanthocephalans, larval and adult nematodes, and an unknown species of helminth. General linear models were used to assess the relationship between host traits and sampling season with parasite infection intensity. The infection intensity of an unidentified plerocercoid species in *P. caeruleopunctatus* was found to be significantly associated with both sampling season and the interaction of host mass with sex. In *P. richardsoni* the infection intensity of the acanthocephalan, *Corynosoma* sp. was found to correlate with sampling season. The highest richness of parasite taxa was recorded from *P. richardsoni*, which may be due to the wide depth range of *P. richardsoni*, coupled with its generalist diet*.*

**Supplementary Information:**

The online version contains supplementary material available at 10.1007/s00436-024-08359-y.

## Introduction

Teleost fish are infected by a wide variety of parasites (Timi and MacKenzie 2015), many of which have major impacts on aquaculture and fisheries (Wood and Lafferty 2015). Multiple studies have sought to identify the factors that influence parasite community composition and richness in teleost fish, including: host size (Guégan and Hugueny 1994; Timi and Poulin 2003), host diet (Lafferty 1999; Luque et al. 2008), host behaviour (Poulin 1999; Sasal et al. 1999), host phylogeny (Braga et al. 2015; Morand et al. 2000), habitat (Klimpel et al. 2006; Timi and Poulin 2003), and seasonality (Poulin and Valtonen 2002; Santoro et al. 2014). There is also evidence to suggest that some of these variables may act synergistically to influence species richness and composition of parasite assemblages in teleost fish (Violante-González et al. 2010).

The effect of host size on parasite richness has been compared to the theory of island biogeography (Poulin 2004; Wilson and MacArthur 1967). Hosts can be seen to function as islands, where the greater surface area available in larger hosts creates more potential niches for parasites (Morand et al. 2000; Poulin 2004). Additionally, the dietary breadth of a host may play an important role in determining the richness of parasite taxa within a given host individual (the infracommunity), through the consumption of a wider range of potential intermediate hosts (Molloy et al. 1995; Locke et al. 2014; Luque et al. 2008). Furthermore, host behaviour such as the gregariousness or propensity to form schools, may exert significant influence on their parasite communities (Sasal et al. 1999). Gregariousness may lead to greater contact between conspecifics, thus increasing transmission rates of parasites, resulting in a greater prevalence of ectoparasitic taxa in the host population (Poulin 1999). Host phylogenetic relationships may also serve to influence parasite community structure in fish (Carrassón et al. 2019; Lima et al. 2012). Furthermore, phylogenetic relatedness can result in taxa sharing similar dietary preferences, behavioural adaptations, and physiology, all of which may result in similar parasite assemblages among related host taxa (Carrassón et al. 2019).

Host habitats can also influence the richness and composition of parasite communities due to a number of abiotic and biotic factors, which operate across a range of temporal and spatial scales, such as the host’s depth range (Luque and Poulin 2004). Greater parasite richness has been observed in teleost fish inhabiting the benthic environment, as opposed to species from the mesopelagic and bathypelagic zones (Klimpel et al. 2006). This may be due to the higher available biomass found in benthic environments which supports a larger number of potential intermediate host species, thus promoting higher parasite diversity (Klimpel et al. 2006; Alves and Luque 2001). The effect of seasonality on parasite communities has also been documented in a number of teleost fish species (Campbell et al. 2007; Ondračková et al. 2004; Poulin and Valtonen 2002; Zander et al. 1999). Seasonal change may act as a stimulus for parasites such as digeneans to seek hosts, as water temperature rises during warmer months, it triggers the release of cercariae from first intermediate hosts, resulting in higher prevalence in downstream hosts (Ondračková et al. 2004).

The investigation of these variables is essential for improving management of fish and their associated parasite communities. This is particularly important for economically important host taxa, so that relevant agencies are suitably equipped in their ecological management (Suthar and Shamsi 2021). One family of teleost fish, the Platycephalidae, commonly referred to as flatheads, are of key importance to both recreational and commercial fisheries in Australia (Gray and Barnes 2015). Platycephalids host a diverse variety of parasite taxa, including, but not limited to a range of ectoparasites such as monogeneans and parasitic copepods, and endoparasites such as digenean trematodes and anisakid nematodes (Hooper 1983; Hossen et al. 2022). Hooper (1983) investigated the parasite communities of seven platycephalid species in New South Wales, Australia, and since then, there have been some investigation into specific parasite taxa from Australian platycephalids (Anderson and Cribb 1994, 1995; Hossen et al. 2021, 2022; Suthar and Shamsi 2021), with a particular focus on potentially zoonotic parasites such as *Echinocephalus* spp. and *Anisakis* spp. (Shamsi 2014; Shamsi et al. 2011, 2021). However, aside from Hooper (1983), none of those studies have quantified the entire parasite community for those platycephalid species in Australia.

In this study, the three species of platycephalids were chosen for investigation based on variations in their respective ecologies, which allowed for the comparison of their parasite community metrics, including prevalence and infection intensity. The first species chosen, the dusky flathead (*Platycephalus fuscus*) inhabits estuarine and near-shore marine environments along the east coast of Australia, ranging between Cairns, Queensland in the north, to the Gippsland Lakes, Victoria in the south (Gray et al. 2002; Gray and Barnes 2015), this is also the largest species of platycephalid, attaining a maximum total length of over 100 cm (Gray and Barnes 2015). The second species included in this study was the blue-spotted flathead (*Platycephalus caeruleopunctatus*). This species of platycephalid inhabits exposed areas of marine sand along the south eastern coast of Australia (Fetterplace et al. 2016; Moore et al. 2011), where it typically occurs in water between 40-100 m deep, however this species is also found in water as shallow as 10 m (Fetterplace et al. 2016; Hossen et al. 2022), and can attain a maximum total length of 62.3 cm (Barnes et al. 2011a). The third species of platycephalid included in this study was the tiger flathead (*Platycephalus richardsoni*), which occurs in offshore marine environments between 40-300 m deep (Fairbridge 1951; Keenan 1991, as cited in Hossen et al. 2021), and can attain a maximum total length of 65 cm (Barnes et al. 2011b).

This study sought to investigate how host traits and environmental factors may influence the parasite communities of three species of Australian platycephalid; dusky flathead (*P. fuscus*), blue-spotted flathead (*P. caeruleopunctatus*), and tiger flathead (*P. richardsoni*).

## Methodology

### Sampling procedure

Three species of flathead were collected over the course of 15 months, between July 2020 and September 2021 (UNE Animal Research Authority No.: AEC19-124). The three species examined in this study were dusky flathead (*P. fuscus*), bluespotted flathead (*P. caeruleopunctatus*), and tiger flathead (*P. richardsoni*). *P. fuscus* and *P. caeruleopunctatus* were caught via angling with hook and line, and euthanised immediately upon capture by pithing the brain case using a pithing tool. This method of euthanasia was selected as it is both humane and effective (Diggles 2016). Specimens were then placed into individual, zip-lock bags labelled with a collection number and site details, including water depth and coordinates. Total length (TL) and mass were recorded for each specimen in millimetres and grams respectively. Both species were collected in the Shoalhaven region of New South Wales, with *P. fuscus* captured from Lake Conjola (within a 5 km radius of the nominated site coordinates: S 35°15.435’ E 150°28.118’) and *P. caeruleopunctatus* collected from an offshore location near Mollymook Beach (within a 5 km radius of the nominated site coordinates: S 35°20.012’ E 150°29.014’). *P. richardsoni* were purchased unfrozen from Fishermen’s Wharf Seafood Pty. Ltd., 1/33 Wason St, Ulladulla NSW 2539, which acquired these specimens through commercial trawl operators working offshore from Eden, NSW.

Depth ranges of specimens collected were 3.2—5.5 m for *P. fuscus*, and 11.4 – 25.5 m for *P. caeruleopunctatus*. Information on depth range was not available for the purchased *P. richardsoni*, as retail staff from Fishermen’s Wharf Seafood Pty. Ltd. could not obtain those records. Based on published literature, the depth range for *P. richardsoni* is typically between 40—300 m (Fairbridge 1951; Keenan 1991, as cited in Hossen et al. 2021). Sampling activities were arranged to allow the collection of host specimens throughout the year to obtain data on potential temporal changes in the parasite communities of the three species of platycephalid.

### Dissection of host specimens and examination for parasites

Necropsy of specimens collected via angling commenced within 1 h of returning from the sampling trip. For *P. richardsoni*, dissection occurred within 30 min after purchase. The external surface and buccopharyngeal cavity were examined for ectoparasites using a hand lens. Sections of dermis, fins, and gills were isolated using a scalpel, and placed into petri dishes of tap water for microscopic examination. Specimens were then dissected, and the internal organs including the liver, gall bladder, pylorus, stomach, intestines, and gonads were separated into petri dishes of water and examined under a stereomicroscope.

**Fig. 1:**
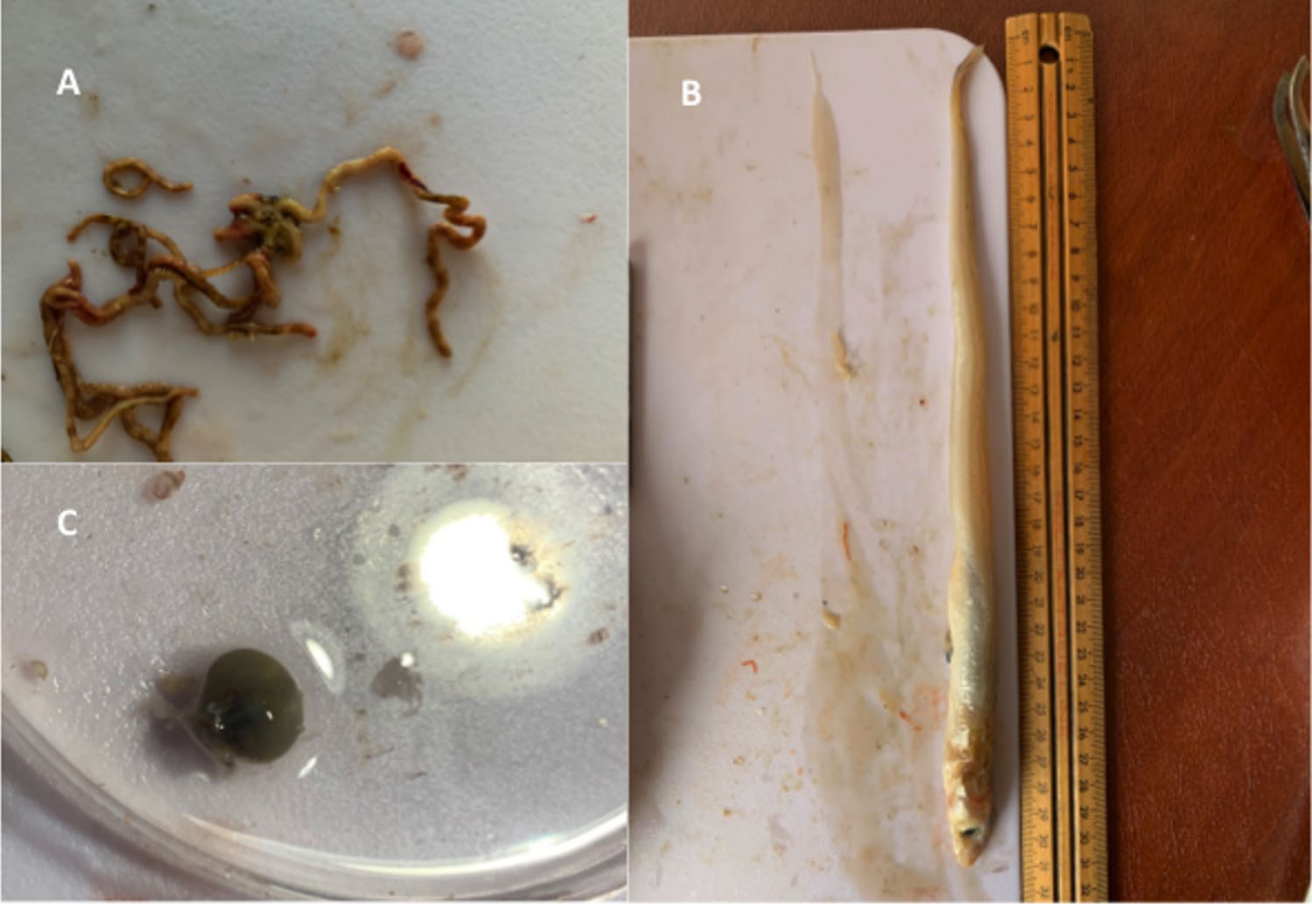
Examples of platycephalid stomach contents retrieved during sampling. Clockwise from top left: **A**) an ophiuroid from *P. caeruleopunctatus*, **B**) a small fish from *P. richardsoni*, and **C**) a brachyuran crab carapace from *P. caeruleopunctatus*

Slices of muscle tissue surrounding the abdominal cavity were examined with a hand lens for the presence of parasites such as anisakid larvae. Parasites were photographed using a microscope to mobile phone camera adaptor (Gosky Optics), and identified via morphological analysis based on Hooper (1983) and other published literature on flathead parasites. Data including abundance, identity, and host site within the fish were recorded for each species of parasite found within a host specimen. The parasites were then removed from the host for preservation in vials of 96% ethanol solution. Stomach contents, and the sex of each fish were also recorded during dissection. Examples of the stomach contents recovered from the host sample populations are visible in Fig. 1.

### Statistical analysis

Statistical analyses were performed using Rcmdr, a GUI for the statistical package R. Due to the small sample size for *P. fuscus* (*n* = 3), only parasite records obtained for *P. caeruleopunctatus* and *P. richardsoni* were used in the data analysis, and only parasite taxa with a prevalence of ≥ 10% were included in the analyses, as parasites with less than 10% prevalence were present in such low number of hosts relative to the total number of hosts sampled, that any analyses on them would not be statistically robust or meaningful. Due to the uneven distribution of parasites among hosts, log transformation of data was performed prior to their use in any statistical analysis. General linear models were generated to assess relationships between parasite infection intensity with host length (mm), mass (g), sex, and sampling season (winter, spring, summer, autumn) as dependent variables.

## Results

### Prevalence and mean intensity of parasites

A total of 14 metazoan parasite species were recorded from a sample of 100 host specimens comprising of three species of platycephalid from the NSW South Coast region. This included one species of copepod, four species of trematodes, one species of cestode, five species of nematodes, two species of acanthocephalans, and one species of unidentified helminth (see Table 1 for details). The number of hosts examined included 3 *P. fuscus*, 38 *P. caeruleopunctatus*, and 59 *P. richardsoni*. The *P. fuscus* sample was comprised of 1 female and 2 male specimens. The *P. caeruleopunctatus* sample was comprised of 1 juvenile specimen of indeterminate sex, 9 female, and 28 male specimens. The *P. richardsoni* sample was comprised of 33 female, and 26 male specimens. The average TL of *P. fuscus* was 368 mm (range = 341-397 mm), and the average mass was 293 g (range = 210-378 g). For the *P. caeruleopunctatus* sample, the average TL was 267.8 mm (range = 197-352 mm), and the average mass was 129.1 g (range = 54-291 g). The *P. richardsoni* sample included the largest hosts measured in terms of TL and mass, where the average TL was 383.7 mm (range = 309-512 mm), and the average mass was 414.5 g (range = 205-903 g). Three species of parasites were recorded from *P. fuscus*, five parasite species were recorded from *P. caeruleopunctatus*, and 13 parasite species were recorded from *P. richardsoni.*

**Table 1:** Taxon, microhabitat, prevalence, and mean intensity of parasites in three Australian platycephalids

		*P. fuscus* (*n* = 3)		*P. caeruleopunctatus* (*n* = 38)		*P. richardsoni* (*n* = 59)	
Taxon	Microhabitat	Prevalence (%)	Mean intensity (range)	Prevalence (%)	Mean intensity (range)	Prevalence (%)	Mean intensity (range)
Crustacea							
*Acanthochondria alatalongicollis*	Buccal cavity	-	-	-	-	8.47 (5/59)	2.8 (1–7)
Trematoda							
*Aponurus* sp.	Stomach	-	-	-	-	18.64 (11/59)	2.09 (1–4)
*Erilepturus* sp.	Stomach	-	-	-	-	5.08 (3/59)	2 (2–2)
*Helicodidymozoon helicis*	Buccal cavity	100 (3/3)	15.67 (5–23)	-	-	-	-
*Lecithochirium* sp.	Stomach	-	-	2.63 (1/38)	1 (1)	10.17 (6/59)	2.5 (1–5)
Cestoda							
Unidentified plerocercoid sp.	Liver	-	-	10.53 (4/38)	3 (1–9)	15.25 (9/59)	1.56 (1–4)
Nematoda							
*Anisakis* sp.	Stomach	-	-	2.63 (1/38)	1 (1)	1.69 (1/59)	1 (1)
*Echinocephalus* sp.	Mesentery	-	-	-	-	1.69 (1/59)	1 (1)
*Hysterothylacium* sp.	Mesentery, stomach	-	-	-	-	10.17 (6/59)	1.5 (1–3)
*Philometra pellucida*	Gonads	66.67 (2/3)	3 (2–4)	5.26 (2/38)	1.5 (1–2)	5.08 (3/59)	1.33 (1–2)
*Raphidascaris* sp.	Mesentery	-	-	-	-	6.78 (4/59)	1 (1)
Acanthocephala							
*Corynosoma* sp.	Stomach, intestines	-	-	94.74 (36/38)	19.81 (3–47)	18.64 (11/59)	24.33 (3–112)
*Raorhynchus terebra*	Intestine	-	-	-	-	5.08 (3/59)	1.5 (1–2)
Unidentified taxon							
Unidentified helminth sp.	Tongue	33.33 (1/3)	1 (1)	-	-	1.69 (1/59)	1 (1)

The parasite with the highest recorded prevalence in *P. fuscus* was the digenean, *Helicodidymozoon helicis*, observed in 100% (3/3) of hosts. For *P. caeruleopunctatus*, cystacanths of the acanthocephalan, *Corynosoma* sp., had the highest prevalence, with 94.74% (36/38) of the hosts infected with a mean intensity of 19.81 ± 1.71 SE (3–47). In *P. richardsoni*, the most common parasites were *Corynosoma* sp. cystacanths and a digenean, *Aponurus* sp., both attained a prevalence of 18.64% (11/59). Of these two parasites, *Corynosoma* sp. had the highest mean intensity, at 24.33 ± 8.62 (3–112) SE. The least common parasites overall were *Anisakis* sp., *Echinocephalus* sp., and an unidentified species of helminth. For *P. caeruleopunctatus*, the least common parasites were *Anisakis* sp. and *Lecithochirium* sp. The parasites with the lowest prevalence in the *P. richardsoni* population were *Anisakis* sp., *Echinocephalus* sp., and a single unidentified helminth of the same species as the one that was found in *P. fuscus*.

**Fig. 2:**
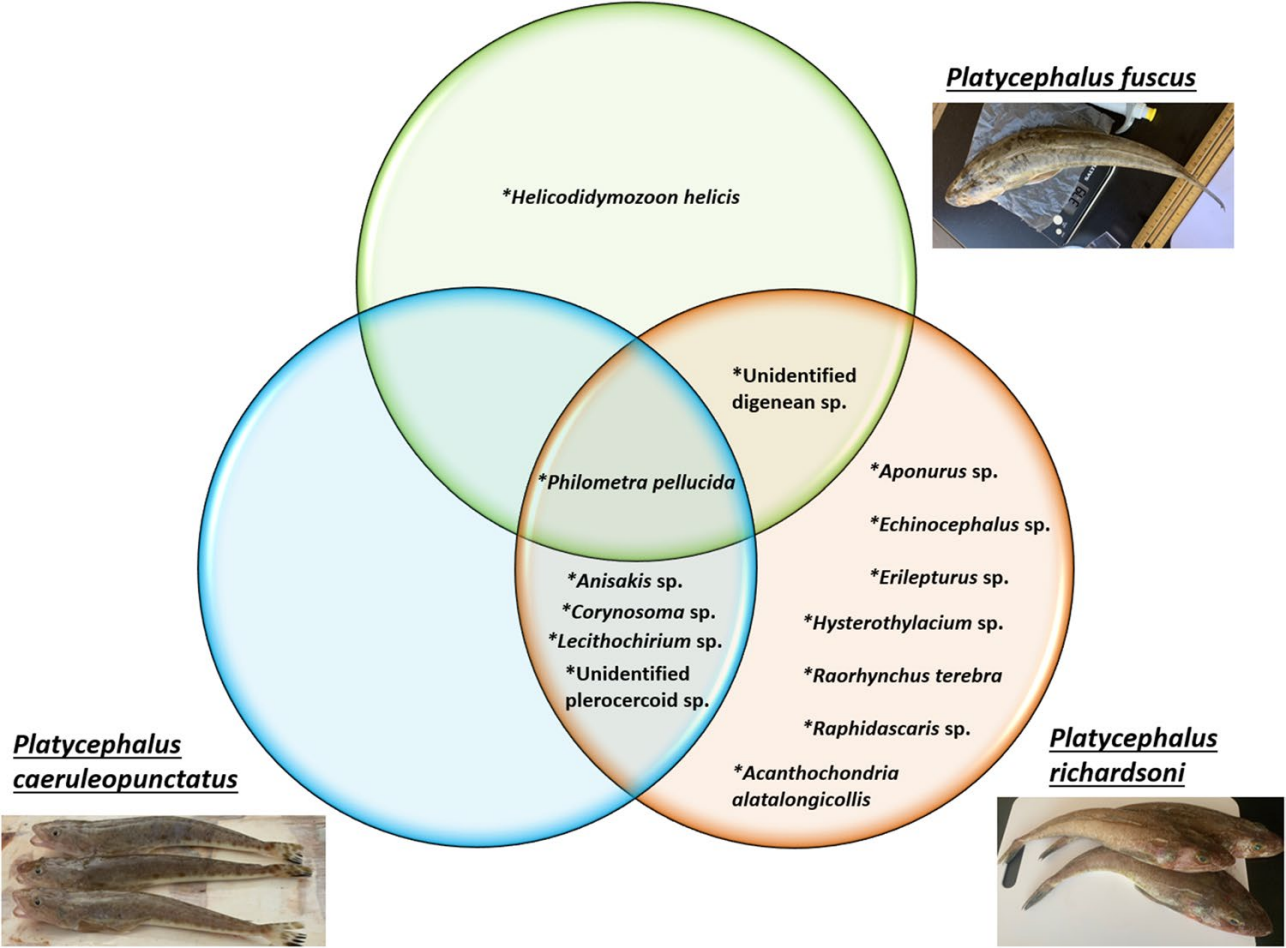
Venn diagram showing the distribution of parasite taxa among three species of Australian platycephalid

Of the three platycephalid species, *P. caeruleopunctatus* and *P. richardsoni* have the greatest commonality in their parasite taxa, with 5 species shared by both host species (see Fig. 2). Only one species of parasite, the nematode *Philometra pellucida*, was found in all host species sampled in this study. The highest diversity of parasites was observed in *P. richardsoni*, which, with the exception of the trematode *H. helicis*, was host to all the parasite taxa that were also recorded from *P. fuscus* and *P. caeruleopunctatus.* This is in addition to 7 parasite species that were only found in *P. richardsoni*. All the parasite taxa recorded from *P. caeruleopunctatus* were also found in *P. richardsoni*. The only ectoparasites recorded during this study was the copepod *Acanthochondria alatalongicollis*, and a species of unidentified helminth, both found in the buccal cavity.

### General linear models

**Fig. 3:**
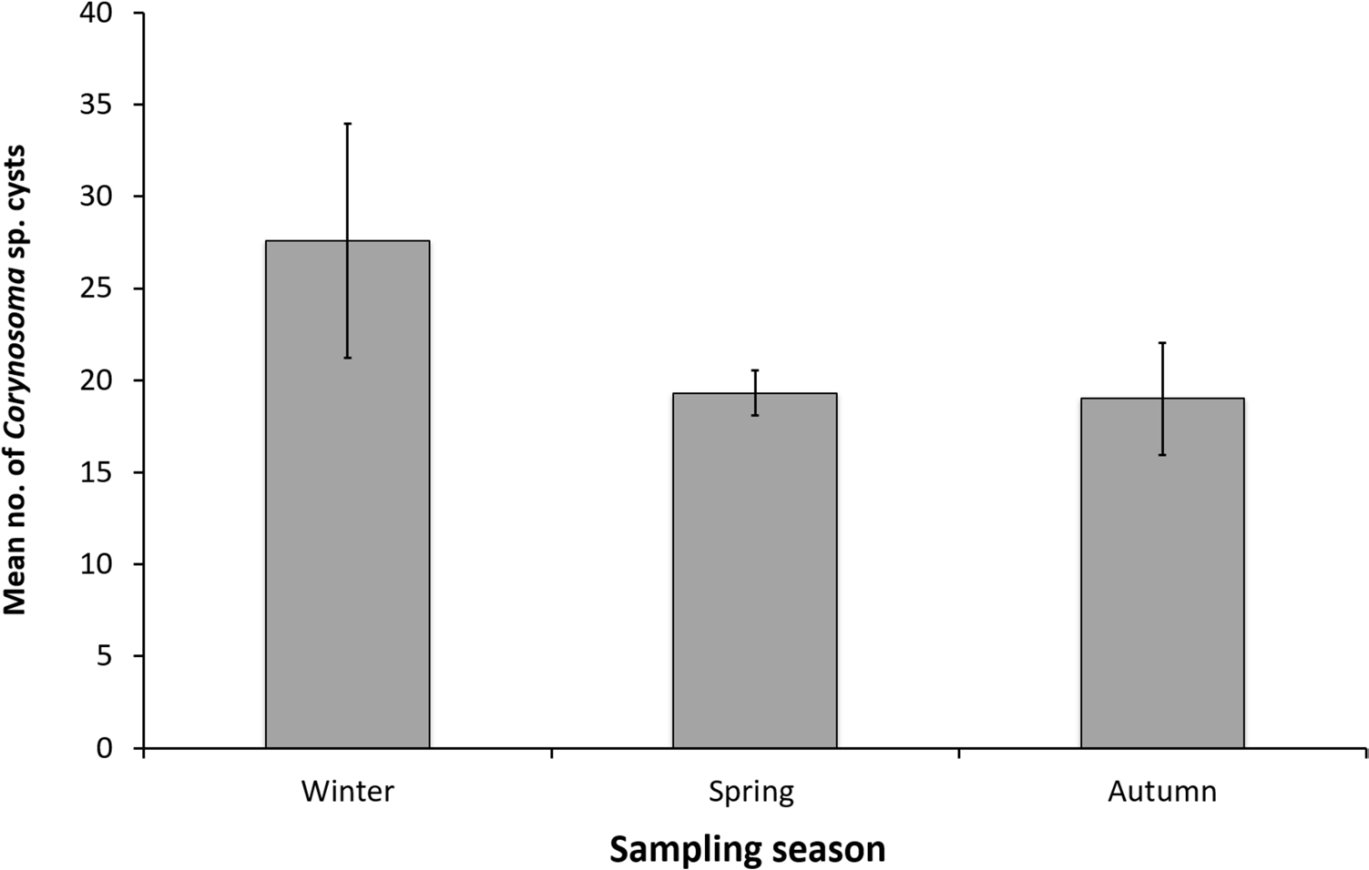
Mean number of *Corynosoma* sp. cysts versus sampling season for *P. richardsoni*

The general linear model analyses for *P. caeruleopunctatus* found a statistically significant relationship between cestode plerocercoids infection intensity and sampling season (*F*_5,31_ = 3.406, *p* = 0.0396), along with a borderline association with mass (*F*_5,31_ = 3.406, *p* = 0.0533), which was found to be more significant once the interaction between host mass and sex has been accounted for (*F*_5,31_ = 3.406, *p* = 0.021). For *P. richardsoni*, *Corynosoma* sp. infection intensity was found to be marginally associated with sampling season (*F*_5,52_ = 1.056, *p* = 0.052, see Fig. 3).

## Discussion

A previous study conducted by Hooper (1983) examining the metazoan parasite communities of Australian platycephalids identified a total of 26 parasite species from a sample of 200 host specimens, comprised of seven species of platycephalids. In the 41 years since that study was published, there has been a small number of other studies focused on quantifying aspects of the infracommunities of Australian platycephalids (Anderson 1998; Anderson and Cribb 1994, 1995; Hossen et al. 2021, 2022; Suthar and Shamsi 2021), however, few have investigated platycephalid parasite communities to the level of metapopulation.

Some of the parasite taxa identified by Hooper (1983), such as the acanthocephalan *Corynosoma* sp. were also recorded in this study. *Corynosoma* sp. was the most frequently recorded parasite in this study, often found encysted along the alimentary canal of both *P. caeruleopunctatus*, and *P. richardsoni*. *Corynosoma* is a cosmopolitan genus that includes over 40 species, and has been recorded in a wide variety of marine and freshwater teleost fish (Hernandez-Orts et al. 2017). For marine species of *Corynosoma*, the acanthella larvae primarily infect crustacean intermediate hosts, such as amphipods, where they develop into a dormant stage referred to as a cystacanth (Hernandez-Orts et al. 2017). Teleost fish that consume infected amphipods serve as paratenic hosts for the cystacanth, which may remain dormant for an extensive period of time (Canel et al. 2019; Hernandez-Orts et al. 2017). When a definitive host such as a marine mammal or sea bird eats infected fish, the cystacanth develops into an adult and undergoes sexual reproduction (Canel et al. 2019; Hernandez-Orts et al. 2017; Weaver and Smales 2014). Given the relatively high prevalence and mean intensity for *Corynosoma* sp. in *P. caeruleopunctatus* and its presence in *P. richardsoni*, this may be used to infer the trophic position of both platycephalids as mid-level predators in their respective food webs, and the inclusion of crustaceans in their diets, with *P. caeruleopunctatus* consuming relatively more crustaceans. This is supported by stomach content recorded in this study, which found a mixture of prey items in both *P. caeruleopunctatus* and *P. richardsoni* that included crustaceans (e.g. Figure 1, see Suppl. Table S1 for details).

In *P. richardsoni*, the relationship between *Corynosoma* sp. infection intensity and sampling season was shown to be marginally significant (Fig. 3). Prevalence and abundance of *Corynosoma* cystacanths in their teleost paratenic hosts can remain remarkably stable across multiple years (Sinisalo and Valtonen 2003), though less is known about potential seasonal patterns. While we found higher infection intensity of *Corynosoma* sp. in *P. richardsoni* during the winter sampling periods, since cystacanths can persist in their paratenic hosts over long durations and accumulate over the fish’s lifespan (Canel et al. 2019; Comiskey and MacKenzie 2000), the flatheads may have acquired the cystacanths during warmer periods months or even years prior to being sampled.

An unidentified species of cestode was found in the *P. caeruleopunctatus* sample, referred to as ‘unidentified plerocercoid’ in this study. Work by Hooper (1983) identified two different types of cestode plerocercoids from platycephalids: one from the family Phyllobothriidae, order Phyllobothriidea, and the other being *Nybelinia* sp., from the family Tentaculariidae, order Trypanorhyncha. Based on the morphology and microhabitat of plerocercoids recorded in this study, it is possible that the unidentified plerocercoid belonged to the family Phyllobothriidae, as it lacks the distinctive hooked tentacles and characteristics of trypanorhynch larvae (Hooper 1983). The order Phyllobothriidea includes species that use elasmobranchs as the definitive host (Caira et al. 2020), which might reflect the mid-level trophic status of platycephalids (as discussed above for *Corynosoma*) through their role as a second intermediate host for larval cestodes.

The infection intensity of this cestode was found to be significantly associated with sampling season along with host mass. However, it must be cautioned that this is based upon a total of four infected hosts from the sample, therefore it difficult to draw any firm conclusions at this point. Furthermore, marine cestode plerocercoids are generally long-lived and their abundance in paratenic hosts are not affected by seasonality (Speare 1995; Braicovich et al. 2012; Pereira et al. 2014). This persistence can lead them to accumulate in paratenic hosts as they age, which may explain the marginally significant relationship that was found between host mass and plerocercoid intensity, as larger, and therefore older fish have been accumulating cestode larvae through consumption of intermediate or paratenic hosts (Poulin 2000). It should be noted that the relationship between host mass and plerocercoid intensity became more significant once the effect of host sex on body mass was accounted for. This might be the result of an uneven sex ratio in the sample population of *P. caeruleopunctatus*, which contained 28 males, 1 juvenile of indeterminate sex, and only 9 females.

Only one species of parasite, the philometrid nematode *P. pellucida*, was found in all three platycephalids, occurring only in sexually mature fishes. Adult philometrids may infect various organs, as well as the visceral cavity and fins in a wide range of freshwater and marine teleost definitive hosts (Iwaki et al. 2020; Moravec et al. 2021; Quiazon et al. 2008; Uhazy 1978). In platycephalids, philometrids such as *P. pellucida* and *P. parabrevicollis* may be found in the gonads (Hooper 1983; Moravec et al. 2021). In this study, the gonads were also recorded as the site of infection for *P. pellucida* in all platycephalid species. Hoopers (1983) reported finding *P. pellucida* in the ovaries of females and the testes of males, and in this study we found mostly female fish with infected ovaries, and a single male dusky flathead that harboured *P. pellucida* in its testes (see Suppl. Table S1). Even though *Philometra* are usually found in the ovaries of female fishes, they have also occasionally been reported from testes of male fishes (Moravec et al. 2014, 2020). The platycephalids in this study may have all become infected with the same species of philometrid through consuming the same type of intermediate host.

Some nematodes recorded during this study may also pose a risk to human health, such as the anisakid nematode, *Anisakis* sp. found in the visceral cavity on the stomach wall of both *P. caeruleopunctatus* and *P. richardsoni*, along with *Echinocephalus*, and *Hysterothylacium* which were found in the mesentery of *P. richardsoni*. *Anisakis* is a widely-recognised zoonotic parasite which uses cetaceans as definitive host and a range of crustaceans, fish, and squid as intermediate and paratenic hosts (Hochberg et al. 2010). *Echinocephalus* uses elasmobranchs as definitive hosts, and a variety of fish and marine invertebrates as intermediate or paratenic hosts (Karagiorgis et al. 2022), and it has been suggested as a potential cause for gnathostomiasis in Australia (Shamsi et al. 2021). The genus *Hysterothylacium* includes approximately 67 species, all of which utilise large teleost fishes as the definitive host and can also accidentally infect humans (Shamsi et al. 2013).

The cystacanths of *Corynosoma* can also be a potential zoonosis as humans may acquire *Corynosoma* infection through the consumption of raw or undercooked fish (Fujita et al. 2016; Sasaki et al. 2019). However, unlike anisakid infections which can cause acute gastrointestinal pain and distress (Hochberg et al. 2010), the symptoms of *Corynosoma* infection are not as severe and may be more chronic in nature (Fujita et al. 2016). As *Corynosoma* are capable of surviving within humans for an extended period of time, an infection by this parasite may lead to cumulative, localized damage at the site of attachment within the intestines (Fujita et al. 2016).

Of the host species sampled in this study, *P. richardsoni* had the highest parasite taxa richness, which may be attributed to the broad depth range of *P. richardsoni*, relative to *P. fuscus* and *P. caeruleopunctatus*, which may have allowed it to encounter a wider range of prey items serving as potential intermediate and paratenic hosts (Luque and Poulin 2004). While the depth range for *P. fuscus* in this study was 3.2—5.5 m, and the depth range for *P. caeruleopunctatus* was 11.4 – 25.5 m, *P. richardsoni* may be found anywhere between 40-300 m (Fairbridge 1951; Keenan 1991, as cited in Hossen et al. 2021). However, depth range alone is not the primary determinant of parasite richness in fish (Luque et al. 2004), and in order to properly evaluate the influence of depth range on the parasite communities of platycephalids, it would be necessary to determine the area covered by the commercial fishing vessel that captured the specimens of *P. richardsoni* as well as the vagility of these fish within the environment.

Most of the parasites found in this study were trophically transmitted and would have been acquired by the flatheads via ingesting parasitized intermediate or paratenic hosts. All four species of digeneans found in the flatheads were adult flukes from the Hemiuroidea superfamily. Species in this superfamily generally have life cycles that involve two to four intermediate hosts, and are trophically transmitted to the definitive predatory fish hosts (Louvard et al. 2024). Some of the other trophically transmitted parasites such as the philometrid *P. pellucida*, found in all host species, may suggest some overlaps in their dietary breadth, as all known philometrid life cycles involves copepods as their intermediate host (Moravec 2004). However, the wide host range of some parasites such as *Anisakis* and *Corynosoma* may limit their usefulness for inferring aspects of host ecology. The developmental stages of parasites found in this study may also be used to infer the host’s trophic position (Poulin and Leung 2011). The presence of both adult and larval parasites in platycephalids suggests that they occupy a mid-level position in the food web, as they are at high enough trophic level to serve as definitive hosts for some parasites, yet also function as either intermediate or paratenic hosts for other parasites (Locke et al. 2014).

Future studies should also include measurements on other variables that can influence parasite richness and intensity, such as host age (Poulin 2000), water temperature, which may affect fish metabolism (Poulin 2020), and salinity, particularly for estuarine hosts (Blanar et al. 2011; Braicovich and Timi 2008; Sobecka et al. 2011).

## Conclusion

Overall, the parasite communities of the platycephalids studied were consistent with previous records documented by Hooper (1983). This suggests that a degree of repeatability exists among platycephalid parasite communities. Hooper (1983) noted that there was very limited information regarding potential predators of platycephalids. This knowledge is still relatively scarce and represents a substantial data deficiency. An investigation of platycephalid prey species and potential predators may allow us to gain a more complete picture of how parasite life cycles relate to the trophic position(s) of these fishes. Finally, investigating other fish and invertebrate species living in sympatry with platycephalids will provide a more comprehensive understanding of parasite communities in the region, and help elucidate the relationship between parasites and trophic interactions in marine ecosystems.

## Supplementary information

Below is the link to the electronic supplementary material.

Supplementary file1 (XLSX 27 KB)

## Data Availability

Data for this study are available from the corresponding author upon reasonable request.
